# The gut dysbiosis-cancer axis: illuminating novel insights and implications for clinical practice

**DOI:** 10.3389/fphar.2023.1208044

**Published:** 2023-06-09

**Authors:** Amer H. Asseri, Tahani Bakhsh, Samah Sulaiman Abuzahrah, Sajad Ali, Irfan A. Rather

**Affiliations:** ^1^ Biochemistry Department, Faculty of Science, King Abdulaziz University, Jeddah, Saudi Arabia; ^2^ Center for Artificial Intelligence in Precision Medicines, King Abdulaziz University, Jeddah, Saudi Arabia; ^3^ Department of Biology, College of Science, University of Jeddah, Jeddah, Saudi Arabia; ^4^ Department of Biotechnology, Yeungnam University, Gyeongsan, Republic of Korea; ^5^ Department of Biological Sciences, Faculty of Science, King Abdulaziz University, Jeddah, Saudi Arabia; ^6^ Centre of Excellence in Bionanoscience Research, King Abdulaziz University, Jeddah, Saudi Arabia

**Keywords:** microbiome, cancer, dysbiosis, tumorigenesis and progression, gut

## Abstract

The human intestinal microbiota, also known as the gut microbiota, comprises more than 100 trillion organisms, mainly bacteria. This number exceeds the host body cells by a factor of ten. The gastrointestinal tract, which houses 60%–80% of the host’s immune cells, is one of the largest immune organs. It maintains systemic immune homeostasis in the face of constant bacterial challenges. The gut microbiota has evolved with the host, and its symbiotic state with the host’s gut epithelium is a testament to this co-evolution. However, certain microbial subpopulations may expand during pathological interventions, disrupting the delicate species-level microbial equilibrium and triggering inflammation and tumorigenesis. This review highlights the impact of gut microbiota dysbiosis on the development and progression of certain types of cancers and discusses the potential for developing new therapeutic strategies against cancer by manipulating the gut microbiota. By interacting with the host microbiota, we may be able to enhance the effectiveness of anticancer therapies and open new avenues for improving patient outcomes.

## 1 Introduction

The gut microbiota comprises a diverse array of commensal microorganisms that reside within the human intestinal tract. The microbiota is primarily composed of bacteria, but also includes fungi, archaea, and viruses, and represents an integral component of the human microbiome (Lynch and Pedersen, 2016). The microbial consortium within the gut, which is heavily populated by Firmicutes and Bacteroidetes, plays a critical role in modulating the host’s metabolism, immunological function, and overall homeostasis (Greenhalgh et al., 2016; Feng et al., 2018). In a state of homeostasis, the gut microbial consortium co-evolves with the host’s mucosal immune system, educating it to tolerate beneficial commensals while limiting the population and infectivity of resident pathobionts within the gut epithelium through a process known as colonization resistance (CR) (Lynch and Pedersen, 2016). CR functions via four primary mechanisms: i) creating a zone of exclusion where the growth of pathobionts is limited through the production of toxic metabolites; ii) modulating the host’s immune system to generate an inhibitory response towards pathobionts; iii) contact proofing through a two-tiered mucus layer on the luminal face and underlying intestinal epithelium; and iv) competitive utilization of limited nutrients available in surrounding zones (Rodríguez et al., 2015; Ubeda et al., 2017; Shen et al., 2021; Tomasova et al., 2021; Yang and Cong, 2021). These mechanisms collectively guard the human gut microbiota against the incursion of pathological infections, highlighting the importance of the gut microbiota in maintaining human health.

The gut-brain axis (GBA) is a complex bidirectional communication system between the gut microbiome and the neuroendocrine and immune systems, which stabilizes the metabolic homeostasis between the host and the gut microbiome (Ortega et al., 2022). The gut communicates its nutritional status to the central nervous system (CNS) through various microbial-produced metabolites, such as the enteroendocrine cells (EECs), the vagus nerve (VN), and the enteric nervous system (ENS), and serves as a communication gatekeeper connecting the gut microbiota with several other organ systems through the CNS (Neuman et al., 2015). Within the gastrointestinal tract, more than 30 peptide hormones are secreted by entero-endocrine cells to regulate digestive processes, gastrointestinal motility, and neurological function (Ceranowicz et al., 2015). As a result, the gut microbiota plays a crucial role in regulating a wide range of gastrointestinal, digestive, and metabolic functions, including the production and assimilation of vitamins, metabolizing dietary compounds, immunity, and protection against gut pathogens invading the body (Vaishnava et al., 2008; Belkaid and Naik, 2013; Carabotti et al., 2015; Magnúsdóttir et al., 2015). Conversely, in a normal or stressful state, the host’s hormones and neuro-hormones may also regulate the gut microbiome composition and metabolites (Ejtahed et al., 2020).

Studies in rodents have demonstrated that gut bacteria can sense several entero-endocrine hormones, such as leptin and ghrelin, which adjust the microbial composition to optimize host health (Ravussin et al., 2012; Queipo-Ortuño et al., 2013). Conversely, the gut microbiota produces active metabolites that are detected by gut cells and then transmitted to the gut-brain axis centers (Sandrini et al., 2015). Commensal gut bacteria produce essential micronutrients, including vitamins K and B, and transform small amino acids into signaling molecules that regulate host metabolism, such as histidine to histamine or glutamate to γ-aminobutyric acid (GABA) (Mohajeri et al., 2018). The *Bacteroides* family synthesizes the anti-diabetic compound such as linoleic acid to catabolize the host’s secondary bile acids and break down phenolic compounds. Furthermore, resident gut bacteria produce hormone-like metabolites, such as short-chain fatty acids (SCFAs; lactate, butyrate, propionate, acetate, and succinate), through bacterial fermentation of dietary fibers in the large intestine (Fukui et al., 2018). These SCFAs are transported through the bloodstream and serve as a primary energy source for the liver. Additionally, SCFAs act as essential signaling molecules for G protein-coupled receptors, including GPR43 and GPR41, that play a role in regulating satiety and increasing energy expenditure (Le Poul et al., 2003). Furthermore, SCFAs regulate glucose and lipid metabolism by affecting intestinal hormone peptide secretion (Clarke et al., 2014).

During the process of feeding, enteroendocrine cells (EECs) that are distributed throughout the gut epithelium get stimulated by nutrient and mechanical stimuli. As a result, they release hormones and neurotransmitters that include serotonin (5-hydroxytryptamine), ghrelin, cholecystokinin (CCK), peptide YY (PYY), and glucagon-like peptide 1 (GLP-1) (Gribble and Reimann, 2016; Worthington et al., 2018). These entero-endocrine hormones exert a range of effects on the gastrointestinal tract, such as regulating the release of hormones like insulin, gastric and bile acids, gut motility, and food intake, which are mediated through vagal afferent neurons or the enteroendocrine system (Chin et al., 2012; Symonds et al., 2015; Bellono et al., 2017).

**FIGURE 1:**
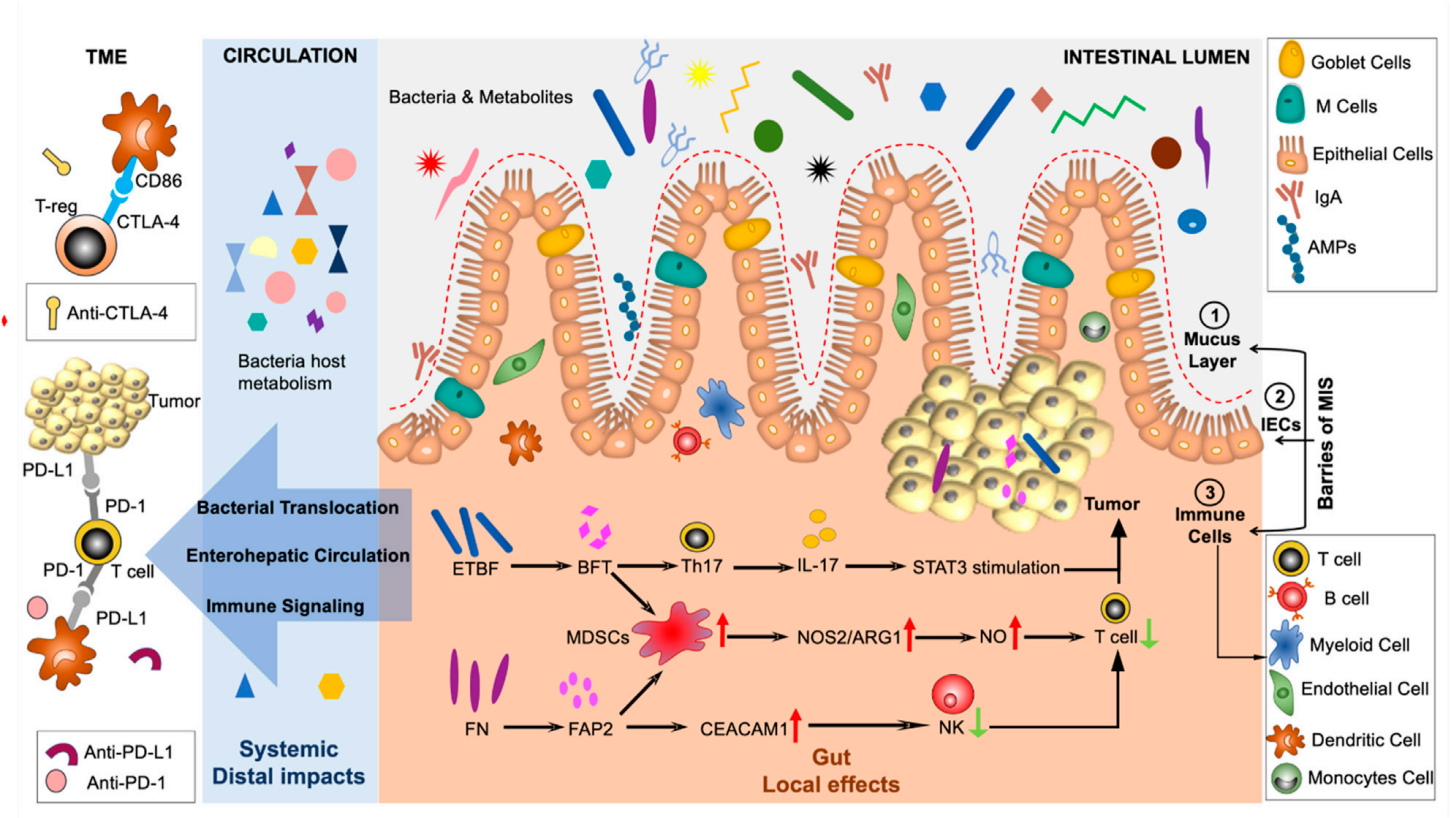
The intricate interplay between the immune response to cancer and the gut microbiota is highlighted in this figure. Mucus serves a pivotal role in safeguarding intestinal cells against the infiltration of microbes. Moreover, effector cytokines and secretory immunoglobulins are crucial components that facilitate the phagocytosis of bacteria. Various factors contribute to the dissemination of immune signals from the gut to remote sites, culminating in immune responses beyond the gut. Abbreviations: CTLA-4, Cytotoxic T-lymphocyte-associated protein 4; MIS, Mucosal immune system; PD-1, programmed cell death 1; IECs, intestinal epithelial cells; ETBF, Enterotoxigenic *Bacteroides fragilis*; CRC, Colitis-associated colorectal cancer; BFT, *Bacteroides fragilis* toxin; TME, tumor microenvironment; iNOS, inducible nitric oxide synthase; NOS2, nitric oxide synthase 2; Mo-MDSCs, monocytic myeloid-derived suppressor cells; NO, nitric oxide; ARG1, arginase 1; FAP2, fibroblast activation protein 2; TME, tumor microenvironment; NK, natural killer; CEACAM1, carcinoembryonic antigen-related cell adhesion molecule 1.

Microbial dysbiosis is characterized by a shift from a diverse bacterial composition to a maladaptive and pathogenic one, and has been linked to numerous diseases, including diabetes, cardiovascular disease, obesity, inflammatory bowel disease, and various cancers (Koren et al., 2011; Karlsson et al., 2012; Karlsson et al., 2013; Kostic et al., 2013a; le Chatelier et al., 2013; Petersen and Round, 2014; Becker et al., 2015; Carding et al., 2015; Franzosa et al., 2019). Carcinogenesis, a multi-step process, is influenced by host immune status and environmental risk factors, of which the gut microbiota and its postbiotics are of prime importance. The gut microbiota produces signaling molecules crucial for developing the host’s immune system (Wu and Wu, 2012). The mucosal immune system (MIS) takes over the front-line defense against pathogen invasion in the host colon. It creates a barrier that helps to keep microbes away from the second layer cells called intestinal epithelial cells (IECs) (Figure 1). The IECs mainly comprise columnar epithelial cells, goblet cells, and M cells that widely express classical pattern recognition receptors (PRRs), such as NOD domain-like receptors (NLRs) and Toll-like receptors (TLRs). These receptors communicate with microorganisms by initially recognizing familiar structures on their surface, such as lipopolysaccharide (LPS), flagellins, bacterial peptidoglycans, and cell wall lipoproteins (de Kivit et al., 2014). The IECs then transduce the signal to intraepithelial lymphocytes to generate effector cytokines that modulate the function on the third layer, called Peyer’s patches and mesenteric lymph nodes residing on lamina propria (Lalani et al., 2020). Comparative studies on germ-free mice have shown that mice with dysbiotic microbiota had disrupted innate and adaptive immune functions, leading to altered immune homeostasis (Kostic et al., 2013b; Weng and Walker, 2013). Studies have also confirmed that commensal and pathogenic bacteria present in the gut have a direct immunoregulatory impact on systemic cancer immunity (Kamada et al., 2013). Cancer cells respond by secreting metabolites that affect the gut bacterial diversity and composition, thereby regulating the tumor microenvironment (TME) and leading to immune inhibition (Ubeda et al., 2017).

## 2 Oncogenesis-dysbiosis relationship

The dysbiosis of gut microbial communities, intestinal epithelium, and the immune system refers to an imbalance in the intricate interactions between these three elements (as illustrated in Figure 1). Gut dysbiosis can lead to inflammation of the gut, neurodegenerative diseases (such as Parkinson’s disease), and cancer due to the emergence of pathogenic populations within the gut microbiota, even at distant sites (Dembiński et al., 2016; Lane et al., 2017; Caputi and Giron, 2018; Yan et al., 2022). Specific pathogens can trigger cancer growth within a dysbiotic gut by negatively impacting the host’s metabolism or gut and immune system functions (Rea et al., 2018). It is worth noting that dysbiotic conditions in the gastrointestinal tract can give rise to tumors both locally and distantly (Sheflin et al., 2014). Microbial pathogens are estimated to drive tumorigenesis in 20% of cases, and microbial commensal imbalance is associated with many types of malignancies (Bhatt et al., 2017). Several preclinical studies using germ-free mouse models have demonstrated that the gut microbiome profoundly influences cancer genesis and progression (Nougayrède et al., 2006; Arthur et al., 2012). According to Li et al. study, overexpression of SQLE, “a rate-limiting enzyme in cholesterol biosynthesis,” has been shown to increase the proliferation of CRC cells by promoting cell cycle progression and suppressing apoptosis with the support of pathogenic bacteria that have been enriched. This study found that compared to control mice stool, Sqle transgenic mice stool increased cell proliferation when transplanted to germ-free mice intestinal barriers. These results suggested that SQLE regulates the gut microbiota-metabolite axis and mediates oncogenesis via cell-intrinsic actions (Li et al., 2022). In another investigation, microbial alterations connected to gastric carcinogenesis’s histological phases were identified. The outcome revealed variations in bacterial interactions throughout the GC stages. Significant enrichments and network centralities point to *P. stomatitis, D. pneumosintes, S. exigua, P. micra*, and *S. anginosus* as potential players in the evolution of GC (Coker et al., 2018). Furthermore, the study meta-analysis revealed extensive and applicable gastric mucosa microbial characteristics related to the histological phases of GC, including the *Helicobacter pylori* effect, GC-linked bacteria, diagnostic biomarkers, and altered bacterial networks (Liu et al., 2022). For the first time, evidence has been found that a bacterial protein known as CagA from *H. pylori* contributes to human cancer (Hatakeyama, 2017). Despite *H. pylori* being the only class I carcinogen listed by the World Health Organization (WHO) (Moss, 2017), a number of studies conducted in cell culture and animal models have investigated the ability of additional microbiota populations to affect DNA replication and integrity (Kim et al., 2002; Toller et al., 2011; Grasso and Frisan, 2015). During pathogenic infections, which cause dysbiosis in the gut microbiome, bacteria can grow and release large amounts of toxins, which then cause DNA breaks in the host, which results in genomic instability, tumor initiation, and progression in those cells that are predisposed to it (Wei Dai, 2014; Frisan, 2016; Zhang et al., 2021). The DNAse activity of colibactin and cytolethal distending toxin (CDT) can be seen in both compounds produced by *Escherichia coli*. Through these DNA double-strand breaks, the toxins can cause transient cell cycle arrests, allow the emergence of genomic mutations, and ultimately lead to tumor development (Lara-Tejero and Galan, 2000). It has also been shown that gut pathogenic bacteria can interfere with DNA damage response and repair pathways, as *Shigella flexneri* does, by causing p53 degradation in host cells by secreting enzymes such as inositol phosphate phosphatase D (IpgD) and cysteine protease-like virulence gene A (VirA), which increases the chances of introducing mutations during DNA damage response (Bergounioux et al., 2012). Furthermore, *H. pylori’s* CagA promotes gastric cancer by interfering with the host’s AKT pathway, causing proteasome-mediated degradation of p53 in gastric epithelial cells. The gut bacteria can also modulate proliferative and survival pathways in the host’s cells, resulting in cancer. Many proteins, such as CagA from *H. pylori*, FadA from *Fusobacterium* *nucleatum*, and MP from *Bacteroides fragilis*, interact with the epithelial E-cadherin of the host (directly or indirectly), disrupting intercellular junctions and activating β-catenin signaling. In turn, the affected host’s cells become proliferating and may undergo cancerogenic transformation (Murata-Kamiya et al., 2007; Wu et al., 2007; Rubinstein et al., 2013).

The *Salmonella enterica* effector avirulence protein A (AvrA) can translocate into the host cells and simultaneously activate the catenin receptor (Lu et al., 2014). A pathogenic infection can potentially induce cancer transformation when it infects pre-transformed cells by releasing other virulence factors in the extracellular gut milieu. In the case of *H. pylori*, CagA is a virulence factor that controls the host’s MAPK pathway. In *S. enterica*, AvrA triggers both the MAPK and the AKT pathways, thus facilitating the host’s survival. *H. pylori* CagA, in particular, can bind many host proteins intracellularly, including SHP-2, a protein tyrosine phosphatase. As a result of CagA-SHP-2 complex formation, SHP-2’s phosphatase activity is deregulated, resulting in the activation of Ras/MAPK signaling.

Furthermore, pathogenic bacteria may indirectly affect tumor development in the human host. A variety of mechanisms can mediate this effect. A major cause of genomic mutations is oxidative stress. It may also involve the development of inflammation or inhibiting the host’s immune response to assist the tumor immunity in escaping. *H. pylori* and *B. fragilis* inject reactive oxygen species (ROS) into the host’s cells, causing hydrogen peroxide and ROS to accumulate and damage DNA. As a result of producing extracellular superoxide, *Enterococcus faecalis* can access the host’s cells through derivative oxygen species. An elevated oxidative environment increases the likelihood of DNA mutations in the host.

## 3 Immunomodulatory axis between the host and the gut microbiota in cancer

A healthy gut microbiome is influenced by the host’s immunity to maintain homeostasis, and conversely, a healthy microbiome contributes to a healthy host*by* influencing the immune system. In addition to influencing gut immunity locally, the gut microbiome affects the immune responses in the distal mucosal sites, which is primarily mediated through systemic metabolic pathways, immunomodulation, and circulatory pathways. The tumorigenicity and host immune system interplay can be categorized into three stages: (a) An elimination state where the tumor is recognized by the host immune surveillance followed by its elimination. (b) An equilibrium state where the immune system can completely eradicate the tumor, nor can it proliferate because of the control over checkpoints via the host immune system. (c) In an immune escape state, the tumor cells evade the immune surveillance and, therefore, proliferate independently of the host immune system (Yang and Cong, 2021; Zhao et al., 2022).

**FIGURE 2:**
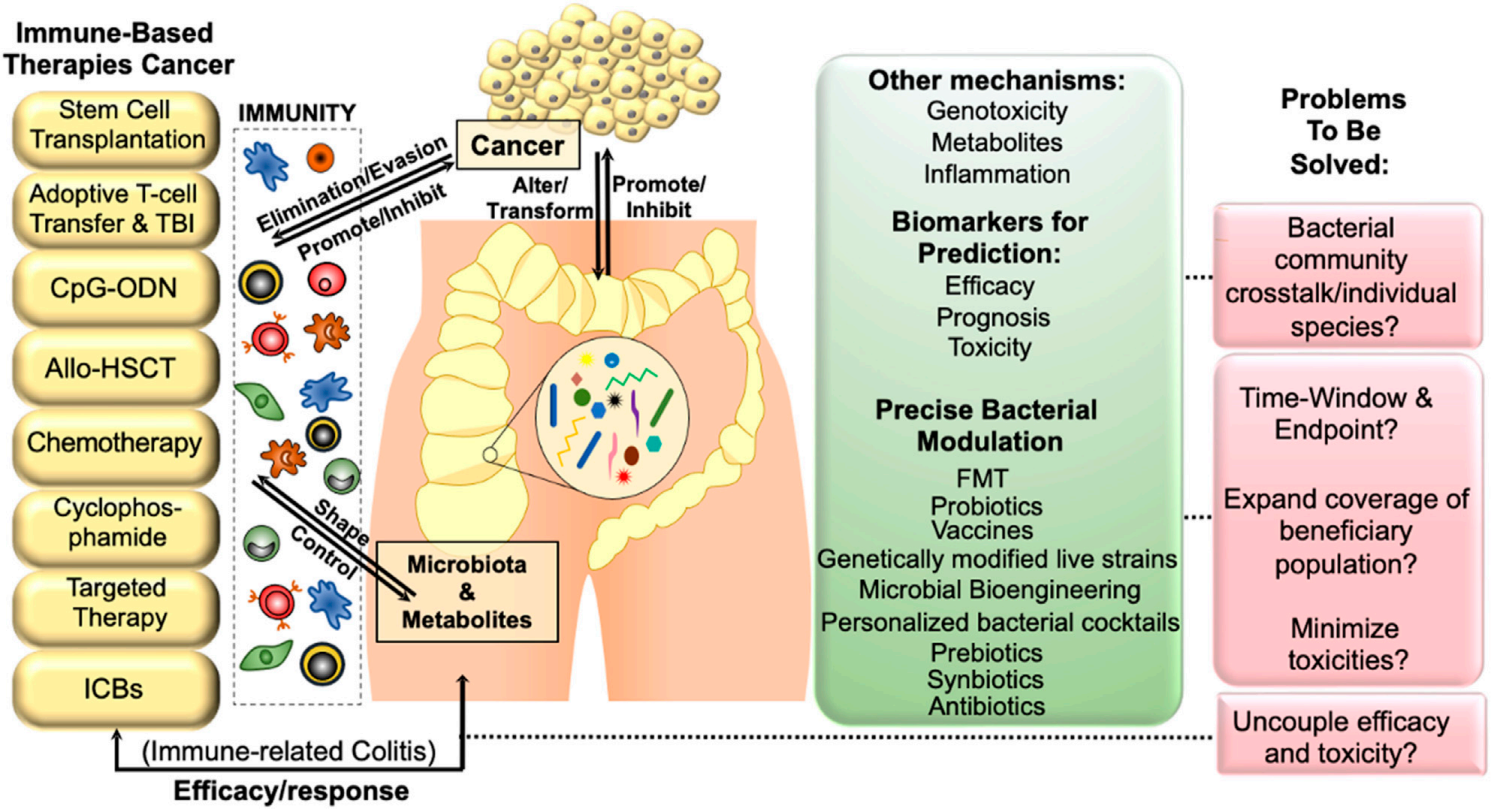
The interplay between gut microbiota and cancer immunotherapy can lead to a more effective treatment strategy. The gut microbiota and its metabolites have a profound impact on the mucosal immune system of the host, ultimately affecting tumor development and progression by modulating immune dysfunction. Host immunity can alter microbial-related signaling and metabolic functions, which may in turn affect tumor surveillance. By promoting or inhibiting immune evasion/elimination, the gut microbiota can influence carcinogenesis, highlighting the importance of manipulating the gut microbiota to improve the efficacy of cancer immunotherapy. Abbreviations: TBI, total body irradiation; CpG-ODN, CpGoligodeoxynucleotides; allo-HSCT, allo-hematopoietic stem cell transplantation; FMT, fecal microbiota transplantation.

Studies on colon tumorigenesis reported that the fecal bacteria *Enterotoxigenic* *B. fragilis* (ETBF), from ApcMin/+ mice, exhibited mucosal dysplasia with increased proportions of T helper (Th) 17 (CD4^+^ IL-17+) and Th1 (CD4^+^ IFN-γ+) cells in lamina propria, thus activating the signal transducer and activator of transcription 3 (STAT3) dependent signalling pathway in colitis-associated colorectal cancer (CRC) (Wong et al., 2017). Studies also showed that the ETBF promoted colon tumorigenesis via a toxin called *B. fragilis* toxin (BFT) and interleukin (IL)-17 in colon epithelial cells through the recruitment of myeloid cells to the tumor microenvironment (TME), which led to the differentiation of myeloid cells into inducible nitric oxide synthase (iNOS) hi monocytic myeloid-derived suppressor cells (Mo-MDSCs). The tumor microenvironment is a micro-niche that surrounds the tumor and includes different types of malignant cells, abnormal vasculature, and immunosuppressive cytokines, which assist the tumor in evading the host immune surveillance (Thaiss et al., 2016; Qiu et al., 2021). The suppressor cells (Mo-MDSCs) upregulated the nitric oxide synthase 2 (NOS2) and arginase 1 (ARG1), therefore, generating protumorigenic nitric oxide (NO) and inhibiting T cell proliferation in the TME (Thiele Orberg et al., 2017) as shown in Figure 1. Colorectal cancer is also associated with the commensal bacteria *F. nucleatum*, which inhibits anticancer T cell-mediated adaptive immunity (Nosho et al., 2016). This study demonstrated that human T cell immunoglobulin and ITIM domain (TIGIT) expressed on natural killer (NK) cells interacting with *F. nucleatum* fibroblast activation protein 2 (FAP2) adhesin interfered with NK cell activity, resulting in the pathogen evading antitumor immunity (Gur et al., 2015; Li et al., 2019). *F. nucleatum* binds to, and induces, carcinoembryonic antigen-related cell adhesion molecule 1 (CEACAM1) expression to inhibit the activities of NK and T cells (Blaser, 2016). *F. nucleatum* also selectively recruits tumor-infiltrating myeloid cells, thus regulating the inflammation in the TME, which is conducive to colon neoplasia. In this regard, MDSCs enrichment and activation significantly promote colorectal carcinogenesis (Kostic et al., 2013a; Cheng et al., 2014) (Figure 2). The local anti- or pro-carcinogenic effects of the gut microbiota translocate from the gut to the distal mucosal sites through the systemic circulation of immune signaling components, microbial metabolites, and enterohepatic circulation.

Recent research indicates that the host’s humoral immunity works bidirectionally in fighting cancer. Innate immunity displays anti-tumor responses by modulating the T-cell immune function or critically shaping the TME (Balachandran et al., 2017). The principal mucosal innate immune cells, mononuclear phagocytes (i.e., monocytes [Mo], macrophages [Macs], and dendritic cells [DCs]) coordinate the immune equilibrium in TME by expressing cytokines that exert immunoregulatory activities (Tait Wojno and Artis, 2016; Qiu et al., 2021). An updated study in 2021 provided insight into the effect of the microbiota on mononuclear phagocytes (MP) in TME. It proposed that the gut commensals can remodel MPs in TME to improve the efficacy of cancer immunotherapy via immune checkpoint inhibitors (ICIs) (Qiu et al., 2021). The group demonstrated that microbiota-derived postbiotic stimulators of interferon gene (STING) such as c-di-AMP activate type I interferon (IFN-I) signaling by intertumoral Mo, which influences the natural killer (NK)-DC crosstalk (Qiu et al., 2021).

Genes encoding NOD1/2, NLRP3, and various toll-like receptors (TLRs) comprise pattern recognition receptors (PRRs) and are expressed on host immune cells such as leukocytes and macrophages (Lam et al., 2021). The PRRs form a central part of the innate immune defence that recognizes pattern-associated-molecular-pattern (PAMPs), including peptidoglycan (PGN), LPS, double-stranded RNA, and CpG DNA as foreign entities which induce signaling cascades involving cytokines and chemokines to help maintain host response against infection (Maloy and Powrie, 2011; Fawkner-Corbett et al., 2017; Keogh et al., 2021). A study showed that a cell wall component LPS expressed by Gram-negative bacteria binds to TLR4, which leads to the induction of nitric oxide and IL-6 production in CRC (Chen et al., 2018). Moreover, the study in CRC mouse models demonstrated that the loss of NOD2 receptor activity, an inflammation and microbiota modulator, led to severe colitis and a higher risk of adenoma (Branquinho et al., 2016). Both studies indicate that modulation of microbiota-dependent innate immune signalling pathways through PRRs could promote host infection, inflammation, and cancer development (Cui et al., 2014).

Furthermore, a study in CRC patients showed that bacterial antigens activated transcription factor 6 (ATF6) during the early gut dysbiosis stages, promoting epithelial barrier damage and innate immune signalling, which triggered tumorigenesis (Coleman et al., 2018). The study confirmed that in nATF6IEC MyD88/TRIF-knockout mice, the bacterial entry in the gastrointestinal tract’s mucus layer induced (MYD88)/TLR adaptor molecule 1 (TRIF)-dependent Stat3 signalling, which led to the tumor promotion (Coleman et al., 2018).

Another study of patients with familial adenomatous polyposis observed a dominant distribution of *B. fragilis* and *E. coli* compared to the controls in the colonic mucosa. The observation was confirmed by the evidence that cohabiting azoxymethane (AOM) and ApcMinΔ716/+ GF mice with enterotoxigenic *B. fragilis* and *E. coli* led to enhancement in IL-17 levels produced by both γδT17 and Th17 immune cells, therefore, increasing the tumor susceptibility (Dejea et al., 2018). In patients with pancreatic ductal adenocarcinoma (PDAC), reports suggest that antibiotics-based bacterial ablation reframed their TME using TLR signaling cascade (Pushalkar et al., 2018). In these PDAC patients, macrophase 1 differentiation was enhanced, MDSC infiltration was reduced, and Th1 differentiation of CD4^+^ T cells and CD8^+^ T cell activation was enhanced.

Conversely, the gut microbiota may also play a crucial role in managing cancer by metabolizing anti-tumor compounds and modulating the immune system and inflammation pathways (Kramer et al., 2018). Iida et al. determined the effect of commensal bacteria depletion in the gut due to a combination of antibiotics, vancomycin, imipenem, and neomycin (ABX) on tumor immunotherapy in tumor-bearing mice (Iida et al., 2013). The tumor-bearing mice underwent a therapy combining intratumoral CpG-oligodeoxynucleotides (ODN), a ligand of Toll-like receptor 9 (TLR9), and inhibitory interleukin-10 (IL-10) receptor antibodies (anti-IL-10R) (Viaud et al., 2011). This combination of immunotherapy retards tumor growth and prolongs the patient’s survival by rapidly inducing hemorrhagic necrosis dependent on tumor necrosis factor (TNF) production by tumor-associated myeloid cells followed by a CD8 T cell response required for tumor eradication (Yang et al., 2013). ABX significantly impaired the therapy efficacy to retard the tumor growth through reduced CpG-ODN–induced TNF expression and decreased the frequencies of TNF-producing cells (Iida et al., 2013). The group also found that the ABX treatment reduced the frequencies of TNF-producing cells and the amount of cytokine per cell in monocytes, macrophages, dendritic cells, and monocyte-derived cells. ABX also diminished the expression of pro-inflammatory Il1a, Il1b, Il12b, and Cxcl10 cytokines (Iida et al., 2013). Thus, the research group concluded that the commensal microbiota peaks the tumor-associated innate myeloid cells for inflammatory cytokine production in response to anti-IL-10R/CpG-ODN treatment, and ABX or the germ-free status of the mice attenuates this response and the TNF-dependent early tumor necrosis. Another study in 2013 also confirmed the role of commensal bacteria in cancer immunotherapy. The tumor-bearing mice were treated with an anti-cancer molecule, cyclophosphamide (CTX), coupled with oral bacterial administration (*Lactobacillus johonsoni* and *Enterococcus hirae*) (Viaud et al., 2013; Daillère et al., 2016). The study exhibited that the coupled treatment led to converting T cells from naïve to pro-inflammatory T helper 17 (TH17), improving the cyclophosphamide efficacy in tumor-bearing mice. In 2015, a group showed that mice bearing lung tumors treated with cisplatin and antibiotics survive less and develop more extensive tumors (Gui et al., 2015). A combinatorial regimen of cisplatin and *Lactobacilli* probiotic strain in tumor-bearing mice resulted in an improved response to therapy. Researchers explained that the combinatorial treatment with a probiotic strain induced a pro-apoptotic cascade within the tumor mass, generating an inflamed necrotic state (Gui et al., 2015). These findings imply that the host’s adaptive immune responses are more specific to antigens, contrary to the host’s diverse innate immune responses, which the gut microbiota could modulate to benefit or harm the host.

## 4 Gut microbiota and cancer immunotherapy

Targeted cancer therapies focus on eliminating specific malignant cells while minimizing the off-target effects that enhance the patient’s overall survival and quality of life (Dy and Adjei, 2013). However, tumor heterogeneity introduces a genetic complexity in malignant tumors. It refers to the divergence of phenotypic and genotypic traits within a primary tumor and its metastases, or between tumors of the same histopathological subtype. Tumor heterogenesis arises when the originating tumor cell, which was the result of a stochastic acquisition of driver mutation/s within the genes, derives a molecularly varied bulk tumor made of multiple clones of the original cancer cell, each one displaying a differential intrinsic sensitivity to the anti-cancer therapies (Bhang et al., 2015). Tumor heterogeneity profoundly influences the type and effectiveness of treatment options available to patients with cancer. It is tightly linked with the development of resistance to therapy or the primary cause of failure of the available anti-cancer treatments, as well as the subsequent tumor relapses (McGranahan and Swanton, 2015). Therefore, understanding the complexity of tumor heterogeneity is critical for developing effective treatment strategies and improving patient outcomes.

The cancer cells are subjected to recognition and elimination by the host’s immune system (Thorsson et al., 2018). In response, the tumor cells constantly evolve to escape such immunosurveillance to expand within the host niche (Thorsson et al., 2018). Alongside chemotherapy and radiotherapy, a novel anticancer approach of targeted cancer immunotherapy is recognized as a significant scientific advance since it can effectively control tumors by rewinding the tumor–immune loop and restoring host antitumor immune responses. Cancer immunotherapy targets cancer resistance and recurrence mechanisms (Emens et al., 2017) and influences patients’ microbiomes, reciprocally affecting their response to such treatments (Roy and Trinchieri, 2017).

The use of the immune system against cancer was initiated with the discovery of immune checkpoint inhibitors (ICIs), mainly due to their bioactivity against histopathologically distinct cancers and efficacy against metastatic tumors (Belkaid and Naik, 2013; Pitt, 2016). However, the clinical response to ICIs based cancer immunotherapy broadly depends on: i) tumor-intrinsic factors such as cell mutational status and oncogenic signaling; and ii) tumor-extrinsic factors such as the TME, metabolic factors, host age and genetics, and environmental factors, microbiota and diet (Park et al., 2018). Inhibitory programmed cell death 1 (PD-1), programmed cell death ligand-1 (PD-L1), or cytotoxic T-lymphocyte-associated protein 4 (CTLA-4) are the key immune checkpoints, and these pathways allow the malignant tumors to evade the host’s immunosurveillance (Marincola et al., 2003); therefore, their suppression activates the immune response against the cancer cells. Therefore, antibodies targeting these checkpoints, such as PD-1 and PD-L1, targetting antibodies (atezolizumab, nivolumab, pembrolizumab, durvalumab, avelumab, toripalimab, sintilimab, and camrelizumab), and CTLA-4 blocker targetting antibodies (ipilimumab), received Food and Drug Administration (FDA) and European Medicine Agency (EMA) accreditation to consider as a standard of care in several advanced cancers such as lymphoma, melanoma, non-small cell lung cancer (NSCLC), prostate cancer, neck cancer, bladder cancer, and kidney cancer (Xin Yu et al., 2019). Clinical trials continue to evaluate their application in adjuvant and combinatorial therapy along with chemotherapy, and other targeted agents against cancers (Barbari et al., 2020).

The gut microbiota has emerged as a critical factor influencing the outcomes of cancer immunotherapy. Numerous studies have demonstrated that specific microbial species are associated with response or non-response to immune checkpoint inhibitors (ICIs), such as anti-PD-1/PD-L1 monoclonal antibodies (Hodi et al., 2010; Topalian et al., 2012; Rosenberg et al., 2016; Gopalakrishnan et al., 2018). For example, certain bacteria, including *Faecalibacterium*, *Bacteroides*, and *Roseburia*, have been found to be enriched in responders to anti-PD-1 mAbs, while others like Ruminococcus are enriched in non-responders (Frankel et al., 2017; Peters et al., 2019). Moreover, fecal microbiota transplantation (FMT) from responders to germ-free mice has been shown to enhance the efficacy of anti-PD-1 mAbs, further supporting the influence of gut microbiota on ICI response (Gopalakrishnan et al., 2018; Fessler et al., 2019; Wilson et al., 2020). A. muciniphila, in particular, has been identified as a potential predictive biomarker for ICI response in NSCLC patients (Derosa et al., 2022). However, there is a lack of consensus on signature species across studies, hindering the establishment of a well-acknowledged consortium of microbial biomarkers. Standardizing methods for sample collection, sequencing, and analysis is crucial to reduce interstudy disparities. Additionally, the integration of multiomics approaches in large cohort studies could provide deeper insights into the correlation between gut microbiota and ICI response.

Modulating the gut microbiota has emerged as a potential strategy to enhance ICI response of patients with non-small cell lung carcinoma (Derosa et al., 2022). Prophylactic antibiotic use should be avoided before ICI initiation, as it has been associated with diminished therapeutic outcomes (Nenclares et al., 2020). Conversely, FMT, probiotics, prebiotics, and dietary modulation hold promise for modulating the gut microbiota to improve immunotherapy response (Ting et al., 2022). FMT has shown efficacy in treating immunotherapy-induced colitis and boosting anti-PD-1 mAbs response in refractory melanoma patients. Clinical trials have demonstrated the safety and feasibility of FMT in cancer treatment, with some patients showing objective responses. However, further research is needed to validate the translational potential of FMT and other modulation methods in immunotherapy. Understanding the interactions among different microbial species and their dominant role in ICI response remains an ongoing challenge.

Therefore, the gut microbiota represents a potential avenue for predictive biomarkers and therapeutic interventions in cancer immunotherapy. The manipulation of gut microbial composition and function could enhance treatment outcomes and improve patient responses to ICIs. However, there are certain weaknesses and strengths that should be acknowledged. One weakness is the lack of standardized methodologies for sample collection, sequencing, and data analysis, which leads to inconsistencies and challenges in comparing results across studies. Additionally, the complexity and dynamic nature of the gut microbiota make it difficult to establish a definitive microbial signature associated with treatment response. On the other hand, the strengths of these studies lie in their use of diverse approaches, including microbial biomarker identification, fecal microbiota transplantation, and manipulation of the gut microbiota through antibiotics, probiotics, and dietary interventions. These investigations have provided valuable preclinical and clinical evidence supporting the influence of the gut microbiota on immunotherapy outcomes.

Moving forward, future studies should aim to address the weaknesses by establishing standardized protocols for sample collection, sequencing, and data analysis. Large-scale, multicenter trials with well-defined patient cohorts would help validate the identified microbial biomarkers and elucidate their predictive value across different cancer types and immunotherapeutic regimens. Longitudinal studies are also needed to understand the dynamics of the gut microbiota during the course of treatment and its impact on long-term response and potential adverse effects. Moreover, further research is warranted to elucidate the mechanisms through which specific microbial communities modulate the immune response and improve immunotherapy outcomes. This knowledge could pave the way for the development of targeted interventions, such as precision microbiota-based therapies, to enhance the efficacy of cancer immunotherapy and improve patient outcomes.

## 5 Nutrition and cancer treatment

Nutrition is considered as one of the major sources to alter microbial structure and function before or during anticancer treatment to improve treatment outcomes and mitigate the adverse effects of microbial alterations during anticancer therapy (Rinninella et al., 2021). Prebiotics like, inulin, fructo-oligosaccharide (FOS), and galactooligosaccharides (GOS), play a major role in promoting the growth of certain group of anaerobic colon inhabiting bacteria (Mithul Aravind et al., 2021). Such compounds are mostly fermented by the bacteria residing in the colon, that efficiently promotes the proliferation of useful bacteria such as Bifidobacterium spp (Gibson et al., 1995; Zwartjes et al., 2021). Enhanced number of Bifidobacterium spp. Have been found to minimize tumor frequency or growth (Reddy and Rivenson, 1993). Perhaps, inulin and oligofructose, is reported to reduce the prevalence of aberrant crypt foci in a study conducted on the colon of rats induced with a chemical carcinogen (Rowland et al., 1998; De Souza-Borges and Conti-Silva, 2018). Another *in vivo* study, demonstrated the impact of 15% inulin and oligo-fructose added into the basal diet of the animals under chemotherapy responses (Taper and Roberfroid, 2005). Both inulin and oligofructose are found to have a significant impact on the therapeutic effect of the anticancer drugs like, vincristine, 5-FU, doxorubicin, cyclophosphamide, cytarabine and methotrexate. Additionally, no adverse impact of the adjuvant therapy of inulin and oligofructose was reported (Taper and Roberfroid, 2005; Witczak et al., 2020).

Moreover, one of the most common complication of enteral nutrition is diarrhea, affecting recovery and leads to extended period of hospital stay, particularly in patients with gastric cancer. One of the study on gastric cancer patients reported that fiber-enriched nutrition formula, and fiber- and probiotic-enriched nutrition formula led to shorter hospitalization period with a minimized diarrhea symptoms (Charteris, 2017). Another study on patients with localized anal canal squamous cell cancer was performed to demonstrate the effect of the gut microbiota and prebiotics during radiotherapy for the effectiveness of treatment and clinical outcomes (Riechelmann et al., 2020). The abundance of Bifidobacterium and *Enterococcus* was increased and with a decrease in the prevalence of *Bacteroides* levels owing to the intake of prebiotic during the preoperative period, while as in the postoperative period, the prevalence of *Enterococcus*, *Bacillus*, *Lactococcus*, and *Streptococcus* raised in the non-prebiotic group. Perhaps, the occurrence of beneficial strains of *Escherichia* and *Shigella* enhanced after prebiotic consumption in the postoperative period. Moreover, prebiotic consumption had significant impact on immunologic indices during the preoperative and postoperative therapy stages (Riechelmann et al., 2020). Significant increase of immunoglobulin (Ig)G, IgM, and transferrin was observed during the preoperative period, and IgG, IgA, suppressor/cytotoxic T cells (CD3^+^CD8^+^), and total B lymphocytes levels in the postoperative period was raised, compared to non-prebiotic group (Riechelmann et al., 2020). In a study on 74 French patients, Ruault et al. demonstrated alteration in biological markers, prior and post 3 months daily consumption of fructo-oligosaccharide. The abundance of Bifidobacterium spp. and *Akkermansia muciniphila* has been noted to have enhanced by inulin (Everard et al., 2013). A latest study indicated that inulin intake results to an enrichment of microbial taxa that enhances the anti-tumor immune system (Fehlbaum et al., 2018). Hence, the adjuvant treatment with inulin and oligofructose could possess significant impact on the effectiveness of cancer chemotherapy via gut microbial modulation and the enhancement in immunity. However, a substantial number of such studies are needed to support and confirm such observations (Mazraeh et al., 2019).

Probiotics are the existing microorganisms in our gut that play a role to healthy status (Sun et al., 2021). Such microbes could be found in fermented foods like yogurt, sauerkraut, and many more (Rezac et al., 2018). In a study of 130 healthy adults, Gonzalez et al. reported the association between the consumption of fermented dairy foods with the daily diet, the gut microbial consortium, and health associated biomarkers (González et al., 2019). It was found that subjects taking natural yogurt had raised levels of fecal Akkermansia, whereas sweetened yogurt consumers were having reduced levels of *Bacteroides*. In another study, probiotic supplementation of patients with Bifidobacterium-containing yogurt product successfully enhanced the prevalence of Bifidobacterium spp. Moreover, in such patients, the prevalence of Barnesiella intestinihominis and Akkermansia muciniphila were also significant (Dizman et al., 2021).

Furthermore, glioblastoma animal models (Maeyama et al., 2021), and early human case studies support the positive effects of the ketogenic diet (high fat, low carbohydrate diet) (van der Louw et al., 2019). Likewise, intermittent fasting and calorie restriction have been demonstrated to alter gut microbiomes and slow cancer progression in animal models. Due to treatment side effects and lack of adherence, human studies on the ketogenic diet and calorie restriction in cancer are challenging. Diet influences cancer treatment outcomes and that specific nutritional and microbial factors positively affect anticancer treatment response (e.g., fasting-mimicking, ketogenic, and high-fiber diets) (Lam et al., 2021; Maeyama et al., 2021; Vernieri et al., 2022). However, we do not yet understand the interaction between diet and microbiome, particularly during treatment. It is most likely a lack of appropriate tools for collecting dietary information, a lack of a suitable study design, a lack of sample size, and the difficulty of working with patients undergoing procedures that result in less quality research (Hughes et al., 2019). Nonetheless, cancer treatment with precision nutrition will remain elusive without these key insights.

To tailor nutrition to individual needs, molecular pathological epidemiology (MPE) is a method that incorporates these factors. There is increasing evidence that germline genetic variations are linked to tumorigenesis, the immune system, and, more recently, the microbiome (Mima et al., 2021). It has been found that there is a 26%–65% heritability of the gut microbiota, according to reports on a study that investigated the impact of genetics on the microbiome using mouse strains (Org et al., 2015). The scientists demonstrated that the gut microbiome is further affected by the interaction between genes and their environment. It was shown that genetic backgrounds had a significant effect on response to a high-fat/high-sugar diet, as well as that the microbiome had a significant effect on regulating metabolism through cross-fostering, in addition to the effects of genetic background differences. There is no doubt that the gut microbiota, along with other studies, can have a distinctive effect on the interaction between genes and the environment, specifically dietary interactions (Touré et al., 2019). Interestingly, MPE studies have also been found to demonstrate diet-immune interactions in the context of cancer, thereby suggesting that people with higher levels of FoxP3+ T regulatory cells (compared to those with low levels of FoxP3+ T regulatory cells) have a decreased risk of colorectal cancer (Song et al., 2016). According to MPE studies, dietary patterns that are prudent with regard to the microbiome are associated with a significant reduction in the development of *Fusobacterium nucleatum*-positive, but not *F. nucleatum*-negative, CRC. *A* low immune infiltration is associated with the microsatellite instability of *F. nucleatum,* which also interacts with tumor genetic features (Hamada et al., 2018). Precision nutrition therapy must consider genetic and environmental factors contributing to the pathology of cancer to enhance the effectiveness of standard cancer treatment.

## 6 Conclusion and future perspective

The paramount influence of gut microbiota on cancer immune response and immunotherapy has given rise to microbiota-based precision medicine as a therapeutic modality in the realm of cancer treatment. Precision therapeutics that are based on the composition of intestinal microflora foster the immune elimination of tumor cells in a more selective and safe manner as opposed to traditional treatments. In addition, the implementation of combined interventions involving antibiotics, prebiotics, probiotics, and postbiotics may increase chemotherapeutic outcomes by altering the gut microbiota. A growing body of evidence supports the crucial role that intestinal microbiota plays in tumor progression, maturation, and therapy response. Therefore, it is conjectured that manipulating the gut microbiota could potentially augment the pharmacological profile of a treatment regimen in cancer patients or reduce the number of specific tumors in the general population. Nevertheless, the complexity of the gut microflora and tumor heterogeneity may pose significant obstacles. However, the application of high-throughput sequencing techniques in conjunction with advanced bioinformatic tools may serve as a feasible approach for enhancing the clinical efficacy of combinatorial anticancer chemo-, radio-, and immunotherapy.
